# Design of PRS enabled monopole slotted-antenna sensor for breast tumor detection

**DOI:** 10.1038/s41598-025-99102-9

**Published:** 2025-06-02

**Authors:** Tiruganesh Lanka, Divya Chaturvedi, M. V. L. Bhavani, Arvind Kumar

**Affiliations:** 1 Department of Electronics and Communication Engineering, SRM University-AP, Amaravati, 522 240 India; 2 Department of Electronics and Communication Engineering, Indian Institute of Information Technology Pune, Pune, Maharashtra 411041 India; 3 Department of Electronics and Communication Engineering, Visvesvaraya National Institute of Technology Nagpur, Nagpur, Maharashtra 440010 India

**Keywords:** Breast phantom, Monopole antenna, Malignant tissues, Partially reflective surface (PRS), Specific-absorption rate (SAR), Cancer, Health care, Medical research, Engineering

## Abstract

This article introduces a near-field microwave sensor based on a monopole antenna for breast tumor detection. The designed antenna operates at 2 GHz and is miniaturized by 37% through the etching of two pairs of rectangular slots along its edges, achieving a compact footprint of 0.28λ_g_ × 0.42λ_g_ (where λ_g_ is the guided wavelength at 2 GHz). To enhance gain, a partially reflective surface (PRS) is positioned behind the radiating monopole, increasing the antenna gain from 2.15 to 7 dBi. The monopole antenna is fabricated using Rogers RT5880 (0.787 mm thickness), while the PRS is made from Rogers TMM13i (3.8 mm thickness). The proposed antenna exhibits high radiation efficiency (95–99%) across the 1.9–2.1 GHz bandwidth. A 3D artificial female breast equivalent phantom is developed to evaluate the sensor’s performance. The PRS-enabled antenna is analyzed in terms of reflected and transmitted power variations at different distances from the phantom. Simulation and experimental results confirm that integrating PRS improves the sensor’s sensitivity and its ability to differentiate between healthy and malignant tissues. Furthermore, specific absorption rate (SAR) analysis indicates that an input power of 50 mW meets SAR safety standards. The proposed antenna is compact, simple, and a safer alternative to X-rays, providing a portable solution for effective breast tumor detection.

## Introduction

Breast cancer stands as a significant cause of female mortality worldwide^1–3^. Detecting breast cancer early significantly diminishes mortality rates, enhancing recover prospects^4–6^. According to current findings, identification of tumors at an initial stage, typically under 5 mm in size, indicates a 97% likelihood of recovery. Prioritizing early detection, not only boosts survival rates but also reduces the necessity for drastic measures like mastectomy^7–9^. A mastectomy is a surgical procedure to remove one or both breasts, typically to treat or prevent breast cancer. Traditional techniques like X-ray mammography, ultrasound, and MRI have downsides such as breast compression, ionizing radiation exposure, high costs, and limited accuracy for specific breast tissue types. Researchers are currently exploring microwave radiation-based systems as alternatives to existing techniques^10–13^. These systems offer several advantages, including lower cost, higher accuracy, and safety from high-energy harmful radiation of X-rays. For instance, microwave imaging (MWI) is a cost-effective, safe, and suitable for wearable or portable devices. Unlike mammography, MWI does not require breast compression or prolonged confinement, potentially offering a more comfortable experience for patients. Moreover, MWI has emerged as a promising alternative, addressing the limitations of ionizing radiation and breast compression^14–17^. Additionally, it is expected to be more cost-effective, as microwave equipment is significantly less expensive compared to MRI systems. These characteristics suggest that MWI could enhance patient adherence in future applications.

Although, MWI remains an investigational technique, it shows promising potential for non-invasive breast assessment by detecting variations in dielectric properties such as conductivity and permittivity, to effectively distinguish between healthy and malignant tissues^18,19^. Further studies are needed to evaluate MWI’s clinical efficiency and diagnostic accuracy relative to established imaging methods. Research indicates that tumors often exhibit variations in dielectric properties, which may be influenced by factors such as water content, cellular density, and tissue heterogeneity. However, these properties can vary significantly across patients and tumor types^20^. Such variability underscores the importance of further investigation into MWI’s diagnostic capabilities in diverse clinical scenarios. The antennas utilized in radar-based imaging systems are typically characterized by several key features, a wide impedance bandwidth, compact size, cost-effective manufacturing, and efficient power coupling to the breast tissues.

Developing a compact breast cancer detection system with the ability to detect deep-seated tumors is highly desirable. While lower frequencies provide greater penetration depth, they require larger sensor sizes, whereas higher frequencies offer finer range resolution but suffer from increased signal attenuation. Numerous efforts have been made to develop compact antennas for microwave breast imaging^21^. One notable advancement is the design of a compact bowtie antenna^22^ operating within the 2–4 GHz frequency range. In this study, the antenna is integrated with a breast phantom composed solely of fat tissues. Another development involves a broadband monopole antenna^23^ covering 3.1–10.6 GHz, though its focus is limited to specific absorption rate (SAR) calculations, varying tumor size and location. Additionally, a two-element dielectric resonator antenna (DRA) sensor array^24^ operating at 6.5 GHz has been introduced for breast tumor detection. However, this study employs a homogeneous phantom with only a fat layer, resulting in minimal sensitivity to tumor presence. Moreover, in^25^, the effectiveness of wide-slot antennas and stacked patch antennas for breast tumor detection is compared. Both antennas are cavity-mounted to improve radiation performance, but their bulky size and the use of a fat-tissue phantom remain limitations. A common challenge in breast cancer detection technologies is that compact near-field antenna sensors typically exhibit lower gain and efficiency, reducing their tumor detection sensitivity. In recent years, partially reflective surfaces (PRS)^41,42^ have gained popularity for enhancing antenna performance, improving bandwidth, gain, and directive radiation patterns while maintaining low cross-polarization^26,27^. For instance, in^26^, a Fabry–Perot cavity-backed antenna achieves a gain enhancement of up to 8 dBi, while in^27^, a large 230 mm × 240 mm partially reflective sheet placed in front of the antenna boosts gain up to 14 dBi through multiple reflections. Similarly, in^28–30^, high-gain antennas are designed using periodic unit-cell arrays arranged in a 2D lattice, combined with basic radiators such as patch, slot, or dipole antennas to further enhance performance.

In this article, a PRS-based monopole antenna-sensor is designed to operate at a frequency of 2 GHz within operational bandwidth of 1.9–2.1 GHz. To achieve a compact-size antenna, four rectangular slots are etched on the non-radiating edges of the patch, leads to 37% reduction in antenna size. The placement of a reflective surface significantly enhances the monopole antenna’s gain from 2.1 to 7 dBi. The PRS is made of sub-wave length unit cells positioned behind the antenna to improve the antenna gain leading to expand sensor’s sensitivity in detecting breast tumors significantly. A heterogeneous female breast equivalent phantom is modeled using CST Microwave Studio and later realistic phantom is fabricated for validation. The proposed antenna sensor demonstrates good sensitivity, achieving high directivity and efficiency. In free space, it provides a gain of 7 dBi, while in a breast phantom environment, it yields a gain of 2.2 dBi.

### Antenna sensor design

**Fig. 1:**
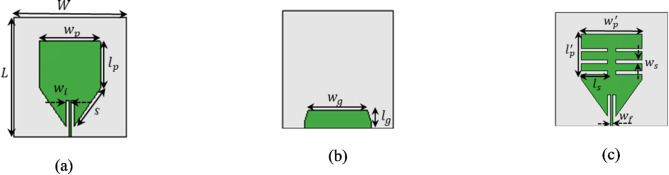
Design evolution: (**a**) Top view of Monopole antenna with *W* = 50, *L* = 50*,* ${w}_{p}$ = ${w{\prime}}_{p}$= 31.5, ${l}_{p}$ = ${l{\prime}}_{p}$  = 23, ${w}_{i}$ = 0.5, *S* = 23, (**b**) Bottom view with ${w}_{g}$ = 28.8, ${l}_{g}$ = 8.5, (**c**) Slotted monopole antenna ${w}_{s}$ = 1, ${l}_{s}$ = 13.6,${w}_{f}$ = 2.6 (units: mm).

The monopole antenna is designed with dimensions including a width *w*_*p*_ and an overall length of *l*_*p*_ + *S* with partial ground plane. The dimensions of monopole antenna are calculated using Eq. (1) operating at a frequency of 2.65 GHz, as shown in Fig. 1a. The ground plane of monopole antenna as shown in Fig. 1b. The operating frequency of the antenna is further adjusted to 2 GHz by etching four rectangular slots of length $\left({l}_{s}\right)$ of 0.2λ_g_ (where λ_g_ is the guide wavelength at 2.65 GHz) along the length edges of the monopole patch as shown in Fig. 1c. The proposed slotted monopole antenna achieves a miniaturization of 37%, offers a fractional bandwidth of 20%, and provides a gain of 2.1 dBi in the operating frequency band 1.83–2.17 GHz. The operating frequency of the monopole antenna excited in its dominant mode is calculated using the following Eq. (1).

1 $$f=\frac{2.2 \times {10}^{11}}{\left(wp+lp+S\right)\times \sqrt{{\varepsilon }_{reff}}}$$

**Fig. 2:**
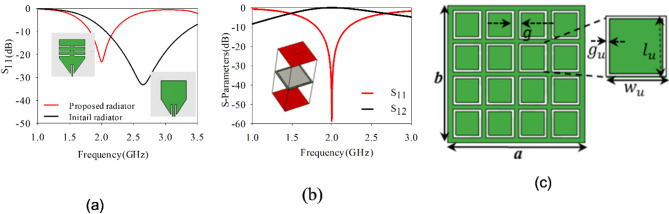
(**a**) S_11_ vs frequency antenna without slots and with slots, (**b**) S-parameter of unit cell, (**c**) array of unit cells, *a* = 50, *b* = 50, *g* = 3, ${l}_{u}$ = 10.3, ${w}_{u}$= 10.3 and ${g}_{u}$ = 0.7 (units: mm).

Figure 2a. demonstrate the S_11_ versus frequency plots before and after incorporating rectangular slots. It reveals that, without slots the monopole radiator resonates at 2.65 GHz in its dominant mode. Carving four rectangular slots along the length of the antenna current, leading to miniaturization and causing the dominant mode to resonates at 2 GHz.

### Design of partially reflective surface

The unit cell is designed by firstly opting a square patch of dimensions *l*_*u*_ × *w*_*u*_ mm^2^ (i.e. 0.22λ_g_ × 0.22λ_g_) and later by etching a ring of width *g*_*u*_ around the boundary of the patch. The unit cell is designed and fabricated on Rogers TMM13i material of thickness 3.8 mm, a dielectric constant of 12.2, and a loss tangent (*tan δ*) of 0. 0019. The unit cell’s dimensions^26,27^ are tuned meticulously to resonate at 2 GHz, as shown in Fig. 2b. The S-parameters of unit cells demonstrating reflection coefficient (S_11_) and transmission coefficient (S_21_), for the proposed unit cell. A 4 × 4 order of unit cells^43–45^ with a uniform gap of *g* between any two-unit cells is created, with overall dimensions of 50 mm × 50 mm, as shown in Fig. 2c.

**Fig. 3:**
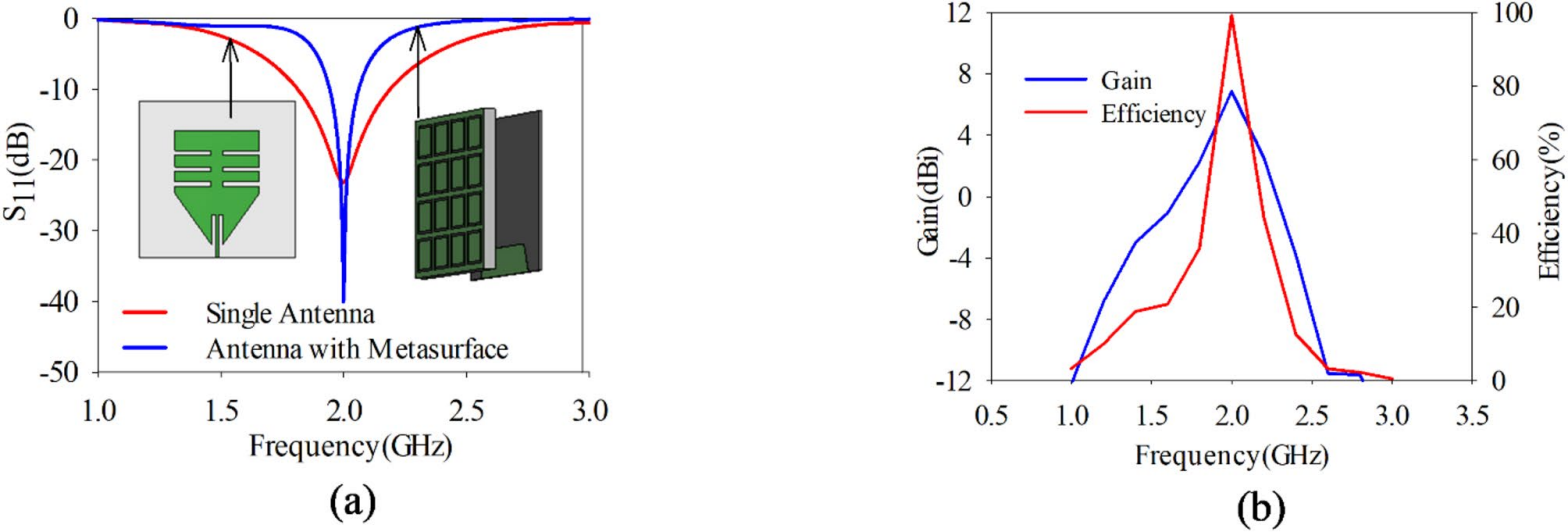
(**a**) S_11_ versus frequency of proposed monopole antenna without and with PRS, (**b**) gain and efficiency versus frequency plot for antenna with PRS.

This array of unit cells is positioned behind the proposed monopole antenna at approximately distance of 0.27λ_g_ (where λ_g_ = $c/f\times \sqrt{{\varepsilon }_{reff}}$, and ${\varepsilon }_{reff}={\varepsilon }_{reff}$ of radiator +${\varepsilon }_{reff}$ of PRS)^31,39^. Figure 3a shows the proposed design performance with and without PRS in terms of bandwidth. By placing the PRS at this distance, it enhances the directional characteristics of the antenna, increasing its forward gain. The reflector helps to focus the radiated energy in the desired direction (i.e. broadside) by reflecting and redirecting the electromagnetic waves in the frequency band of 1.9–2.1 GHz. Moreover, it improves the front-to-back ratio of the antenna by significantly reducing the amount of radiation in the direction opposite the intended beam. Figure 3b presents the gain and efficiency characteristics of the proposed antenna sensor. Upon integrating the PRS, the sensor exhibits an increased gain nearly 7 dBi, while achieving an efficiency of up to 99%. The incorporation of the PRS leads to a 12% decrease in the fractional bandwidth of the sensor. The 4 × 4 grid creates a symmetric structure that promotes a balanced and uniform reflection pattern.

**Fig. 4:**
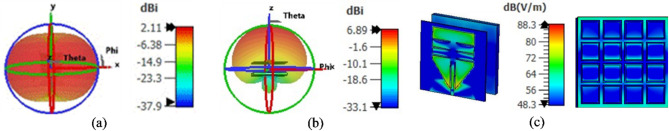
(**a**) 3D radiation pattern of a proposed slotted monopole antenna, (**b**) antenna with PRS, (**c**) Scalar electric field distribution on antenna and PRS.

This symmetry helps the antenna maintain a stable and consistent radiation pattern, which is essential for detecting tumors accurately in breast tissue by avoiding any unintended lobes or nulls in the radiation field. As shown in Fig. 4a, the single antenna emits an omnidirectional radiation pattern with a gain of 2.15 dBi. In contrast, when equipped with PRS as illustrated in Fig. 4b, the antenna’s gain is enhanced by 4.85 dBi, resulting in a unidirectional radiation pattern. In Fig. 4c the top metallic layer of both the patch and PRS, displaying only the scalar electric field (E-field) at 2 GHz to focus on understanding the magnitude of the E-field distribution. Although, it is chosen to illustrate the scalar field here, it is also possible to analyze the vector E-field at this frequency to observed amplitude as well as phase for a more comprehensive view.

### Antenna- sensor performance over heterogeneous breast phantom

To assess the efficiency of the suggested sensor identifying breast tumor cells, the antenna-sensor is simulated using CST Microwave Studio 2022 near a 3D heterogeneous hemispherical breast phantom mimicking female breast. A hemispherical phantom is fashioned with four distinct layers: skin, fat, fibro glandular, and tumor. The dielectric characteristics of the breast tissues at 2 GHz operating frequency utilized in this study are outlined in Table 1, sourced from references^35,36^.

**Table 1:** Electrical properties of breast tissues at 2 GHz^35,36^.

Tissues	Relative permittivity (${\epsilon }$r)	Conductivity (σ) (S/m)	Density (ρ) (kg/m^3^)	Tissues layer thickness (mm)
Skin	38	3.6	1010	4
Fat	11	0.2	928	16
Fibro Gland	18	0.8	1030	30 (r)
Tumor	56	3.8	1100	8 (r)

*r* = radius of the respective tissue.

**Fig. 5:**
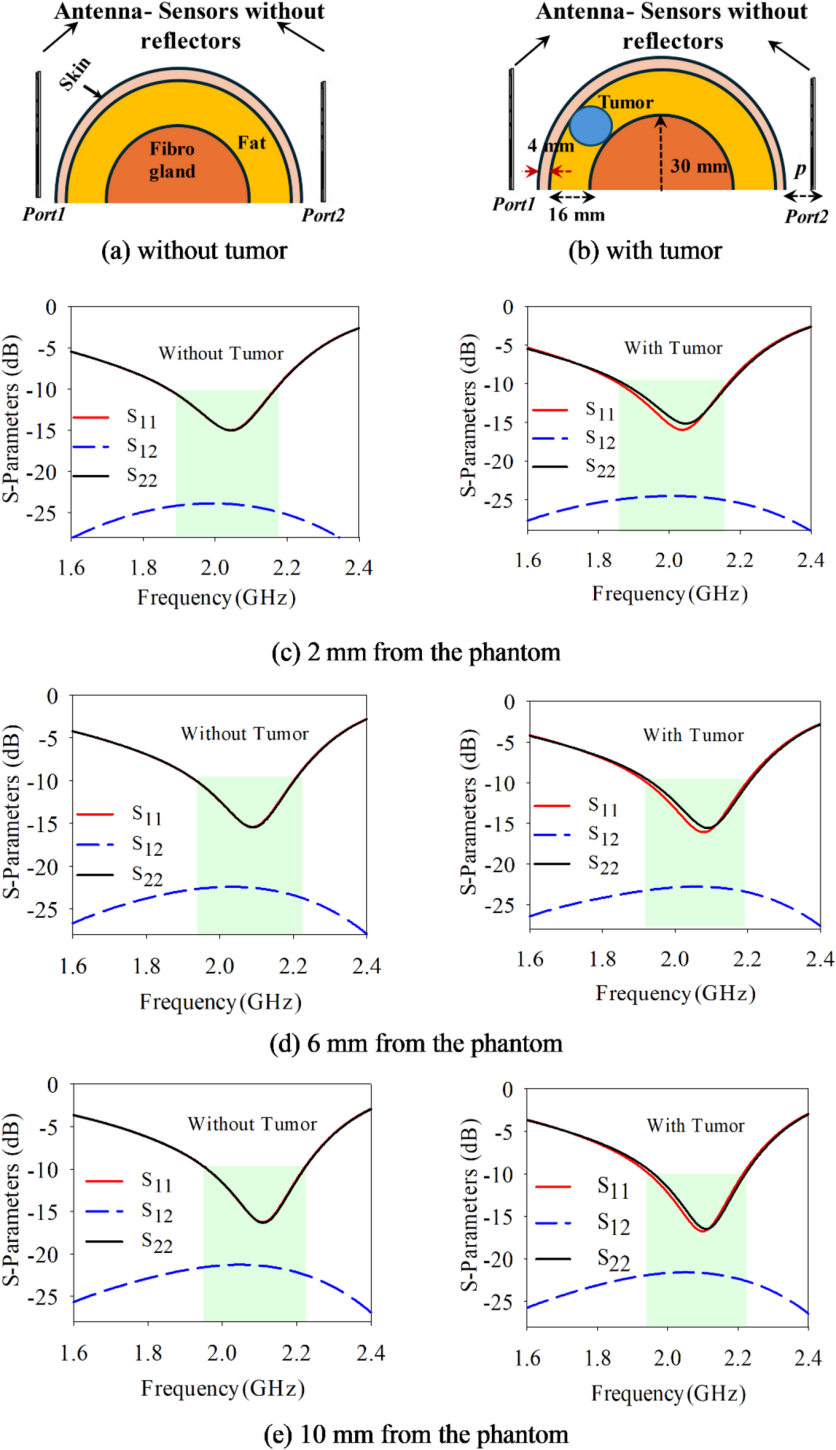
Antenna-sensors without reflectors positioned at different distances from the phantom.

The dielectric properties including permittivity (*ε*_*r*_) and conductivity (*σ*) are analyzed at 2 GHz using a one-pole Cole–Cole model as referenced in^15,35^. In all simulation studies, the tumor’s location within the fat layer is kept consistent and the tumor’s radius is maintained fixed of 8 mm. Fig. 5 illustrates the S-parameters variation with placement of the antenna-radiator without PRS at a distance *p* mm, from a breast phantom. Figure 5a shows the S_11_ variation in operating frequency band without tumor while Fig. 5b shows it with tumor. The thickness of skin and fat layers of heterogeneous phantom has been considered 4 mm and 16 mm, while the radius of the fibro-gland layer and tumor are considered 20 mm and 8 mm, respectively. The S-parameters are obtained for both conditions of healthy tissues and tissues containing malignant tumors as mentioned in Table 2. Figure 5c shows the variation in S-parameters at a distance of 2 mm from the phantom. It shows, the reflection loss of around -15 dB at an operating frequency of 2.024 GHz in healthy case (i.e. without tumor) while a small deviation of 1 dB between reflection loss (S_11_) and (S_22_) at 2.024 GHz in the case malignant tumor. Similarly, at 6 mm, in healthy case, the reflection loss of around − 15 dB at an operating frequency of 2.028 GHz while in tumor case there is 1 dB variation at 2.028 GHz, as depicted in Fig. 5d. Moreover, at 10 mm, in healthy case, the reflection loss of around − 15.5 dB at an operating frequency of 2.11 GHz while in tumor case there is − 16.2 dB variation in reflection losses at *port1* and *port2* at 2.11 GHz, as depicted in Fig. 5e. It is quite evident in Fig. 5 at different distances of sensor from phantom that the sensitivity of detection of tumors is limited. These figures demonstrate a minor variation in S-parameters with tumor presence while small variation in frequency can be observed at different distances from phantom.

**Table 2:** S-Parameters/frequency response of antenna sensors without PRS.

Tissues	Case	S11 (dB)	*fr*	S12 (dB)	S22 (dB)
Healthy	Case 1 (2 mm)	− 15.2	2.024	− 23.4	− 15.2
Tumor		− 15.1	2.024	− 24.2	− 16.2
Healthy	Case 1 (6 mm)	− 15.5	2.07	− 22.4	− 15.5
Tumor		− 16.2	2.07	− 22.7	− 15.6
Healthy	Case 1(10 mm)	− 16.2	2.11	− 21.3	− 16.2
Tumor		− 16.7	2.11	− 21.7	− 16.3

**Fig. 6:**
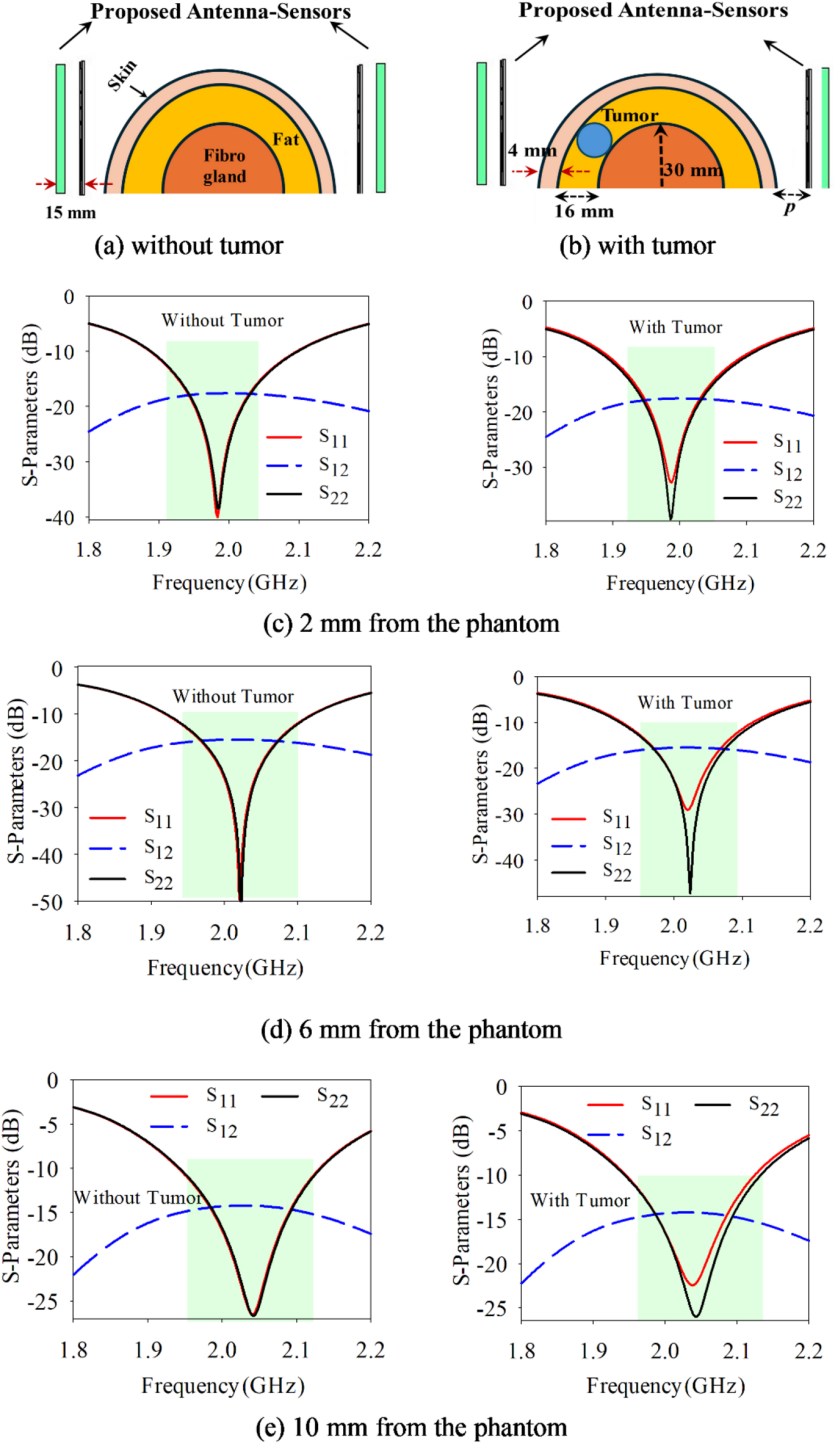
Proposed Antenna-sensors with PRS positioned at different distances from the Phantom.

The similar analysis is performed by placing antenna-sensor with PRS near to the phantom to detect the presence of tumor. The respective breast equivalent heterogenous phantoms are shown in Fig. 6a and b, respectively with healthy case and malignant case. When the proposed antenna-sensor is placed at 2 mm distance from phantom in healthy tissues both S_11_ and S_22_ are observed around − 38 dB and operating frequency of 1.99 GHz, as mentioned in Table 3.

**Table 3:** S-parameters/frequency response of antenna sensors with PRS.

Tissues	Case	S11 (dB)	*fr*	S12 (dB)	S22 (dB)
Healthy	Case 1 (2 mm)	− 38.2	1.99	− 17.3	− 38.1
Tumor		− 32.5	1.985	− 17.1	− 38.8
Healthy	Case 1 (6 mm)	− 49.4	2.02	− 15.4	− 49.7
Tumor		− 28.6	2.02	− 15.5	− 47.1
Healthy	Case 1(10 mm)	− 26.5	2.04	− 14.25	− 26.5
Tumor		− 22.2	2.04	− 14.35	− 25.8

However, in the presence of tumor the S_11_ is observed of − 32.5 dB while S_22_ is observed of − 38.8 dB at 1.986 GHz shown in Fig. 6c. Similarly, when both antenna sensors moved away to 6 mm from breast, in healthy case both S_11_/S_22_ are observed around − 50 dB with resonating frequency of 2.02 GHz while in presence of tumor, the S_11_ is observed of − 28.6 dB and S_22_ is observed of − 47.1 dB at 2.02 GHz shown in Fig. 6d. Further, when both antenna- sensors are moved at 10 mm from the phantom, in healthy case the S_11_/S_22_ are observed of − 26.5 dB at 2.04 GHz while in the presence of tumor the S_11_ is observed of − 22.2 dB and S_22_ is observed of − 26.4 dB at 2.04 GHz shown in Fig. 6e. From the S-parameters study, it can be concluded that the resonating frequency varies primarily with the location of the antenna sensors, while significant differences in the magnitude of S_11_ and S_22_ values indicate the presence of a tumor. The use of PRS greatly enhances the sensor’s ability to detect contrasts in dielectric properties between healthy and malignant tissues, leading to successful tumor detection. With PRS, the antenna sensor’s sensitivity is notably improved, achieving a gain increase from 0.931 to 2.95 dBi when close to the breast phantom.

### SAR evaluation and other important parameters

**Fig. 7:**
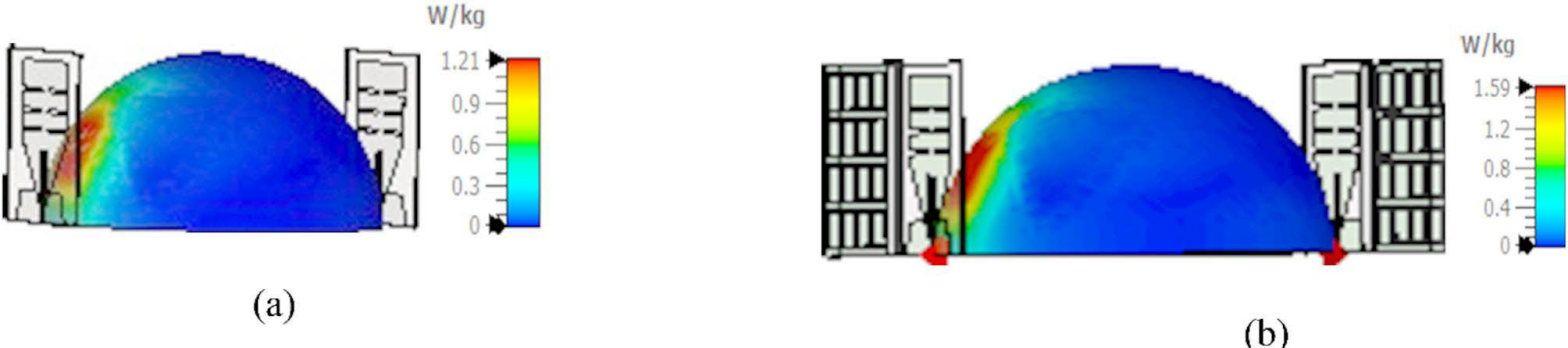
SAR at 2 GHz (50 mW input power) over 1 gm mass of the tissues at 2 mm distance from phantom (**a**) with proposed slotted monopole antenna, (**b**) antenna with PRS.

The specific absorption rate (SAR) is calculated to quantify the rate at which the human body absorbs electromagnetic energy^16^. According to the IEEE C95.1 standard^32^, the SAR must not exceed 1.6 W/kg for 1 gm mass of tissue to adhere to safety guidelines for radiation exposure. The SAR is assessed for both cases: with a single antenna radiator and with the antenna enabled with PRS. The SAR value for the single antenna is 1.21 W/kg, while with PRS, it increases to 1.59 W/kg, though it remains within safe limits shown in Fig. 7a and b. To ensure compliance with SAR regulations, an input power of 50 milliwatts was used for both scenarios. Despite the notable increase in directive gain with PRS, the SAR value remains only slightly higher and still within safe thresholds. Moreover, the lossy nature of breast tissues affects the antenna’s frequency response due to power absorption across various layers. The penetration of EM waves into tissue layers is essential for identifying dielectric contrasts. The penetration depth determines how far the waves can effectively travel within each tissue layer, aiding in assessing the sensor’s sensitivity to detecting tumors. The penetration depth depends on the skin depth factor, which varies with the dielectric properties of each layer, leading to differing levels of absorption and scattering. Based on loss tangent the material can be classified as good conductor, good dielectric and lossy dielectric. The loss tangent and penetration depth can be evaluated by using the following Eqs. (2) and (3)^34,35^

2 $${\text{Loss }}\;{\text{tangent }} = \tan \delta = \frac{\sigma }{\omega \varepsilon } = \frac{\sigma }{{\omega \varepsilon_{o} \varepsilon_{r} }}$$

if ($\frac{\sigma }{\omega \varepsilon }\gg 1$) represents the good conductor or $(\frac{\sigma }{\omega \varepsilon }\ll 1)$ exhibits the good dielectric medium. The corresponding values of loss tangent have been displayed in Table. 4, that confirm the different tissues are lossy dielectrics. The penetration depth in lossy dielectrics is determined by the attenuation constant (1\α) from the relation^34^

3 $$\delta \, = { 1}/\alpha$$

where

**Table 4:** EM wave parameters in breast tissues at 2 GHz.

Tissues	Loss tangent $\left(\sigma /\omega \varepsilon \right)$	Attenuation constant $\left(\sigma \right)$(Np/m)	Skin depth $\left(\updelta \right)$(mm)
Skin	0.85	101.88	9.96
Fat	0.163	11.19	89.36
Fibro Gland	0.4	34.79	28.74
Tumor	0.610	91.51	10.93

4 $$\alpha (\text{Nepers}/\text{m})= \omega \sqrt{\frac{\mu \varepsilon }{2}\left(\sqrt{1+{\left(\frac{\sigma }{\omega \varepsilon }\right)}^{2}}-1\right)}$$

## Experimental results

**Fig. 8:**
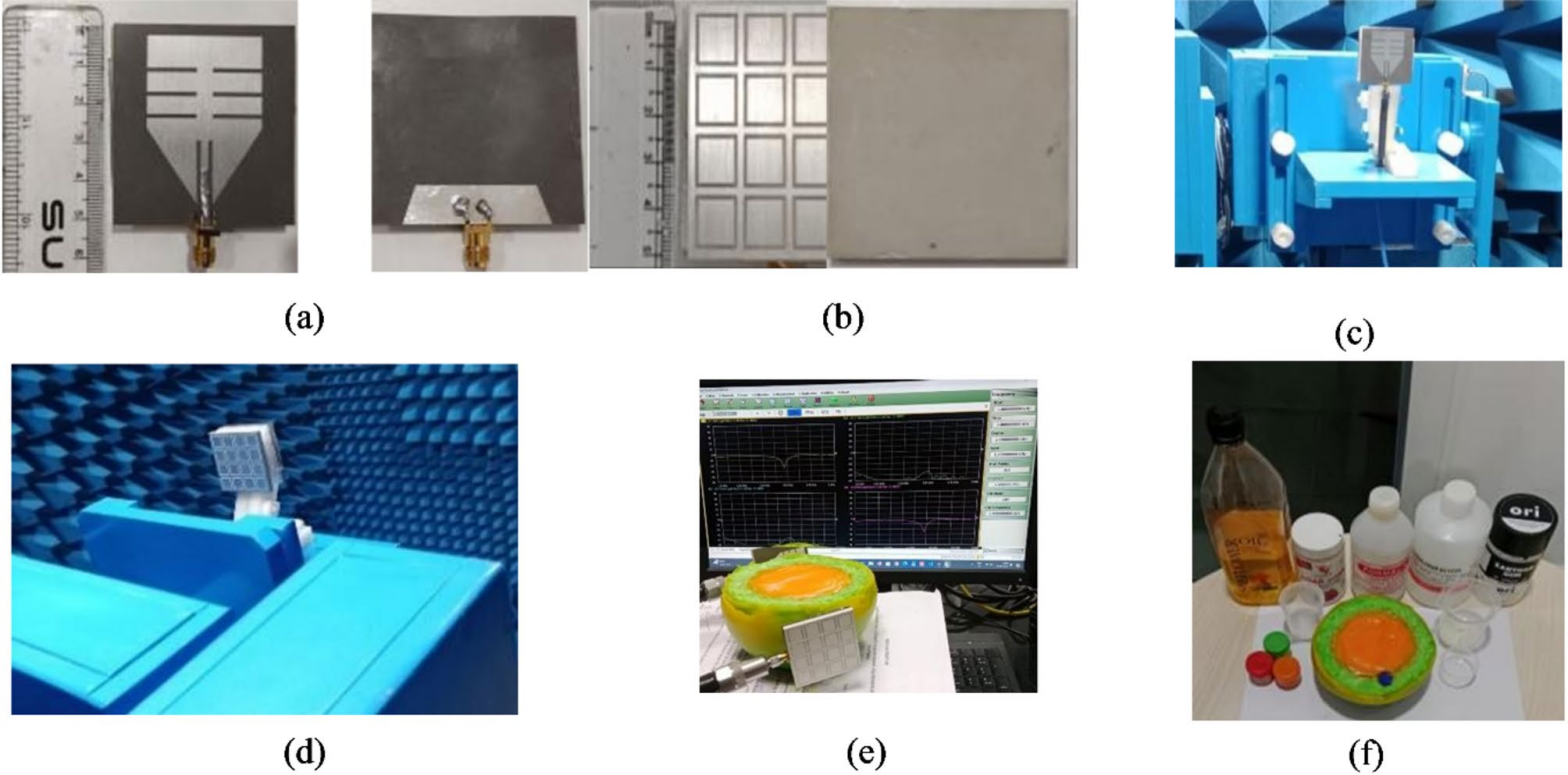
Fabricated prototype (**a**) top and bottom view of monopole antenna, (**b**) top and bottom view of PRS, (**c**) and (**d**) proposed antenna with PRS under test, (**e**) Heterogeneous breast phantom without tumor, (**f**) Phantom with tumor.

To validate the experimental outcomes, the proposed antenna-sensor is fabricated on an RT Duroid 5880 substrate, featuring a dielectric constant of 2.2, a loss tangent of 0.009 and a thickness of 0.787 mm by using conventional PCB process. Additionally, the PRS is constructed on a Rogers TMM 13i laminate of thickness 3.8 mm of dielectric constant of 12.2 and a loss tangent of 0.0019. The PRS is placed towards the ground plane of antenna with the help of foam material of dielectric constant 1.1. The fabricated prototype’s top and bottom views and antenna under test are presented in Fig. 8. The top- and bottom-plane of monopole radiator is shown in Fig. 8a, while Fig. 8b shows the planes of PRS. The proposed PRS enabled antenna sensor is tested in an anechoic chamber to evaluate the gain and radiation patterns as shown in Fig. 8c and d respectively.

**Fig. 9:**
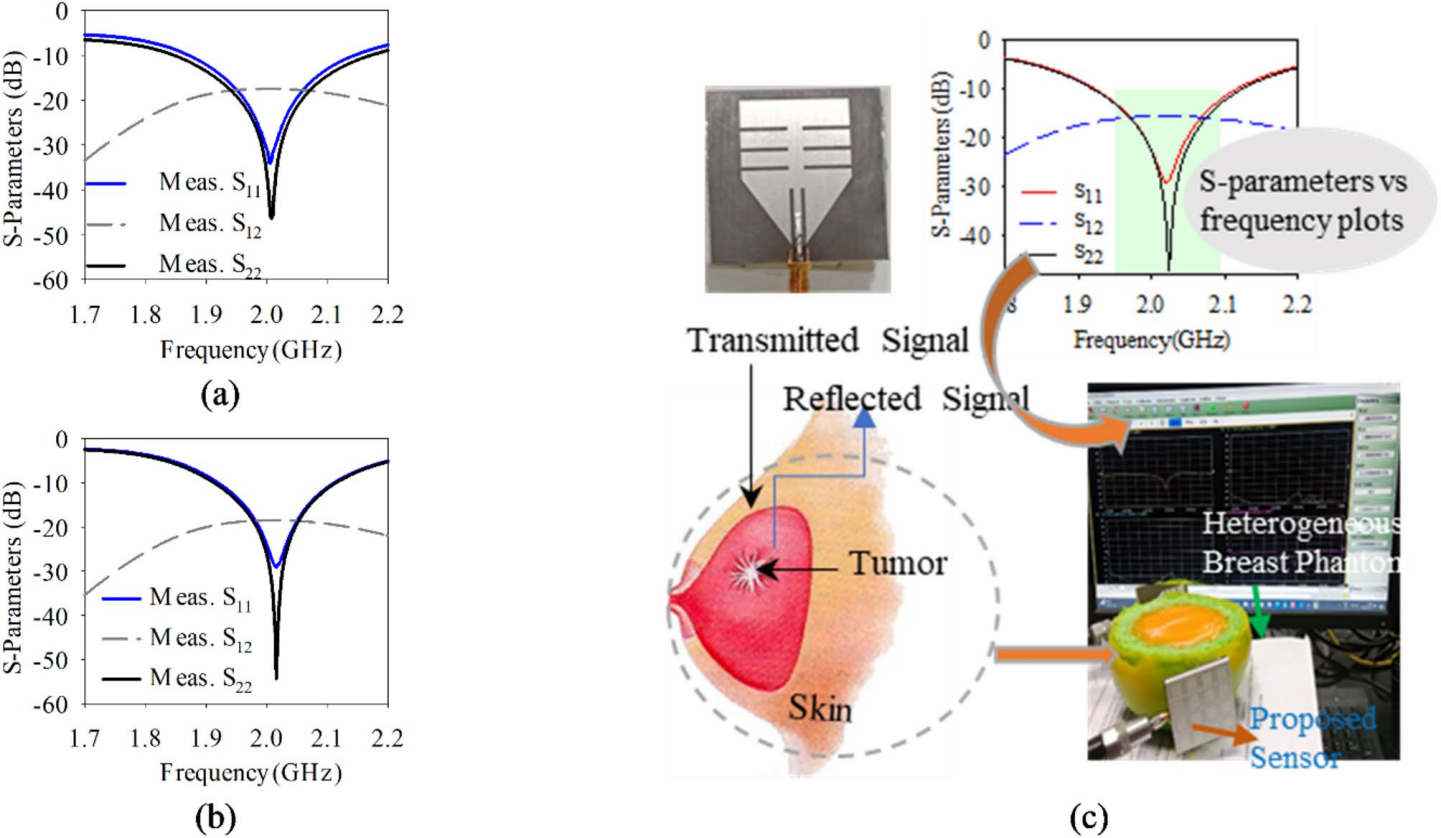
(**a**) The measured S_11_/S_22_/S_12_ versus frequency plots 2 mm distance from phantom, (**b**) antenna-sensor placed at 6 mm, (**c**) set up for tumor detection using proposed antenna-sensor.

A realistic heterogeneous breast phantom equivalent to female breast tissues is developed using natural materials, such as distilled water, agar powder, propylene glycol, safflower oil, ensuring preservation of the dielectric properties of skin, fat, fibro-glandular tissues, and tumor. The detailed methodology and composition of material in preparing these heterogeneous breast phantoms with and without tumor are comprehensively discussed in^33,34^. Later, the dielectric constant is measured for each homogeneous layer the help of MS46122B shock line Vector Network Analyzer (VNA) using dielectric resonator method^35^. The radius of overall phantom is considered 50 mm with dimensions are considered same of simulation studies. The corresponding images of these realistic phantoms can be seen in Fig. 8e and 8f respectively. To ensure the performance of the fabricated antenna-sensor, it is tested for S-parameters along with PRS at two different distances 2 mm and 6 mm. Figure 9a depicts the S_11_/S_22_/S_12_ vs frequency response when antenna-sensors are placed at 2 mm away from the breast phantom. The S_11_ is observed of − 38 dB while S_22_ observed of − 48 dB at 2.015 GHz. When antenna sensors moved away further and placed at a distance of 6 mm from the breast, the S_11_ is observed of − 28.8 dB and S_22_ is observed of − 52 dB at 2.02 GHz shown in Fig. 9b. Thus, it is quite evident from the measurements results that a significant difference in the magnitude of S_11_ and S_22_ are observed in the presence of tumor. Figure 9c, illustrates the antenna-senor setup used for identifying the tumor presence.

**Fig. 10:**
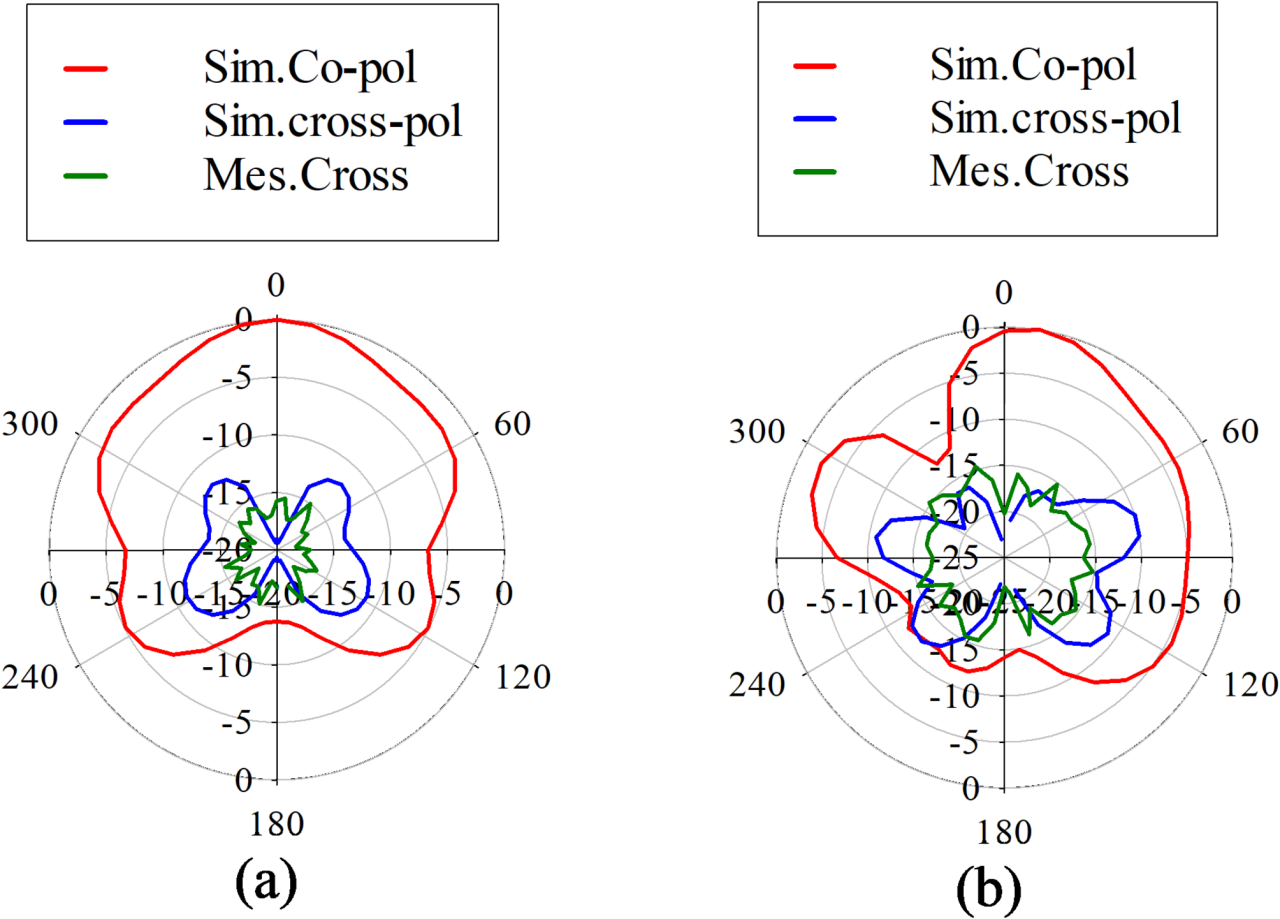
2D radiation pattern plots at 2.02 GHz in free space (**a**) at ϕ = 0° and (**b**) ϕ = 90^o^.

Figure 10 illustrates the simulated and measured 2D radiation patterns of proposed PRS enabled monopole antenna at a frequency of 2.02 GHz at two principal cut-planes (ϕ = 0°) and (ϕ = 90°). These plots reveal that the proposed antenna exhibits a unidirectional radiation pattern, attributed to the reflective surface is positioned on the backside of the monopole antenna. The cross-polarization level is measured 20 dB below the co-polarization level in both principal cut planes in the broadside direction, and a front-to-back ratio (FTBR) of 14 dB is observed in the same direction. Table 5 provides a comparative analysis of the proposed antenna-sensor parameters with those of existing designs in the literature. The proposed antenna stands out for its simplicity in sensors is capable to detect the presence of malignant tissues based on its dielectric contrast sensing ability, making it easily functional and effective for tumor detection. The uniqueness of the proposed antenna-sensor lies in its simplicity and high detection ability that can detect the tumor’s presence near the fibro glandular layer due to its low operating frequency 2 GHz and high directive gain.

**Table 5:** Comparison of the proposed sensor with previous works.

References	Bandwidth (GHz)	Size of Sensors (a*λ1* × b*λ1*)	No. of sensors used	Antenna type	Gain (dBi)	Compact/portable	Tumor detection method	Measurements were conducted using heterogeneous breast phantoms that were prepared in the lab (Yes/No)
^1^	2.4–2.45	0.83 *λ*1 × 0.83 *λ*1	1	Textile Antenna	3	Portable alone	Sensing	No
^8^	1.54–7	0.80 *λ*1 × 0.68 *λ*1	1	Vivaldi antenna	9.8	Portable alone	Imaging	Only homogeneous phantom is used
^9^	2.5–8.5	2.13 *λ*1 × 2.64 *λ*1	1	Cavity-backed Vivaldi	2.1	Neither compact nor Portable	Sensing	Only homogeneous phantom is used
^15^	1–1.3	0.85 *λ*1 × 0.20 *λ*1	4	Dipole array sensor	Not specified	Portable alone	Sensing	No
^16^	1.1–1.3	0.85 *λ*1 × 0.34 *λ*1	4	Loop Array sensor	Not specified	Portable alone	Sensing	No
^21^	3.5–15	2.80 λ1 × 3.34*λ1*	4 × 4	Slot Antenna	Not specified	Compact alone	Imaging	Only homogeneous phantom is used
^22^	1.8–4.1	0.72 *λ*1 × 0.72 *λ*1	1	Bowtie Antenna	Not specified	Neither compact nor portable	Imaging	No
^23^	3.7–7.0	0.75 *λ*1 × 0.69 *λ*1	1	Monopole microstrip antenna	Not specified	Portable alone	Sensing	No
^37^	7.98–8.14	0.53 *λ*1 × 0.53 *λ*1 (size of single element)	24	Patch antenna	1.04	Portable alone	Imaging	No
^38^	3.6–9.2	0.65 *λ1* × 0.54 *λ1*	1	Microstrip Patch	Not specified	Portable alone	Imaging	No
^41^	2–14	0.55 *λg* × 0.55 *λg*	4	Microstrip patch antenna	11.7	Portable alone	Imaging	No
^42^	7.8–15	0.47 *λg* × 0.41 *λg*	1	Slotted Triangular Flared (STF) patch	6	Neither compact nor portable	Imaging	No
Prop. Sens	1.9–2.1	0.28 *λ1* × 0.42 *λ1*	2	PRS Enabled Monopole Slotted Antenna Sensor	7	Compact and Portable	Sensing	Yes

Where λ1 is the guide wavelength at dominant mode resonance.

Unlike many previous studies, this work includes experimental validation using a realistic heterogeneous breast equivalent phantom, closely mimicking the properties of actual breast tissues. The results demonstrate a clear distinction in return loss between the normal breast tissue and the presence of a tumor. Additionally, a consistent agreement is observed between the experimental findings and the simulated results, further confirming the reliability of the proposed design. Moreover, the proposed one offers advantages of miniaturized design that allows for ease of use in clinical settings, a critical advantage over bulkier sensors. Also, it offers a high efficiency of 95–99% across the 1.9–2.1 GHz frequency range, thus it minimizes power loss, ensuring that a high proportion of the input power is directed toward tissue interaction. The proposed sensor setup utilizes two compact antenna-sensors for breast tumor detection, offering a simple yet effective design that enhances portability. Despite its straightforward construction, the system provides a clear distinction between normal and malignant tissues with the help of S-parameters. Optimal matching is maintained without the need for any matching medium or lumped loads when the sensor is in proximity of the tissue.

## Conclusion

In this study, we present a novel near-field microwave monopole antenna-sensor incorporating a partially reflective surface (PRS). The PRS-enhanced sensor has a compact 50 × 50 × 18.8 mm^3^ size, operates at 2 GHz. By integrating the PRS, the sensor achieves higher directive gain, significantly improving its ability to detect deep-seated tumors within breast tissue. The sensor’s effectiveness is demonstrated through numerical simulations, which detect variations in a breast phantom. Validation is performed through measurements on an artificially created heterogeneous phantom, designed to replicate breast tissue properties. A strong correlation is observed between simulated and measured S-parameters within the 1.9–2.1 GHz frequency range. This antenna-sensor offers a viable, non-invasive solution for breast tissue characterization and accurate tumor detection, demonstrating its potential for early-stage breast cancer diagnosis. While the study provides promising results, certain parameters require further exploration. Future research will focus on developing a low-cost, portable breast cancer detecting system using proposed antenna sensor. A detailed investigation will be conducted to analyze power dissipation and energy distribution across different layers of the breast phantom. Understanding microwave energy absorption at varying depths will help optimize the sensor design for improved sensitivity and signal penetration in real-world clinical applications. Furthermore, specific absorption rate (SAR) analysis will be expanded to evaluate the impact of tumor positioning within the breast phantom. Once the system is fully developed and verified for SAR compliance, clinical trials will be conducted in an authorized hospital following all ethical approvals and safety regulations.

## Data Availability

The datasets used and/or analyzed during the current study available from the corresponding author on reasonable request.
